# Novel DNA Biosensor for Direct Determination of Carrageenan

**DOI:** 10.1038/s41598-019-42757-y

**Published:** 2019-04-23

**Authors:** Riyadh Abdulmalek Hassan, Lee Yook Heng, Ling Ling Tan

**Affiliations:** 1 School of Chemical Sciences and Food Technology, Faculty of Science and Technology, University Kebangsaan Malaysia, 43600 UKM Bangi, Selangor Darul Ehsan Malaysia; 2 Department of Chemistry, Faculty of Science, Ibb University, P.O. Box: 70270, Ibb, Republic of Yemen; 3 Southeast Asia Disaster Prevention Research Initiative (SEADPRI-UKM), Institute for Environment and Development (LESTARI), Universiti Kebangsaan Malaysia, 43600 UKM Bangi, Selangor Darul Ehsan Malaysia

**Keywords:** Screening, Target identification

## Abstract

A novel disposable electrochemical biosensor based on immobilized calf thymus double-stranded DNA (dsDNA) on the carbon-based screen-printed electrode (SPE) is developed for rapid biorecognition of carrageenan by using methylene blue (MB) redox indicator. The biosensor protocol for the detection of carrageenan is based on the concept of competitive binding of positively charged MB to the negatively charged dsDNA and carrageenan. The decrement in the MB cathodic peak current (*i*_pc_) signal as a result of the released MB from the immobilized dsDNA, and attracted to the carrageenan can be monitored via differential pulse voltammetry (DPV). The biosensor showed high sensitivity and selectivity to carrageenan at low concentration without interference from other polyanions such as alginate, gum arabic and starch. Calibration of the biosensor with carrageenan exhibited an excellent linear dependence from 1–10 mg L^−1^ (R^2^ = 0.98) with a detection limit of 0.08 mg L^−1^. The DNA-based carrageenan biosensor showed satisfactory reproducibility with 5.6–6.9% (n = 3) relative standard deviations (RSD), and possessing several advantages such as simplicity, fast and direct application to real sample analysis without any prior extensive sample treatments, particularly for seaweeds and food analyses.

## Introduction

**Figure 1:**
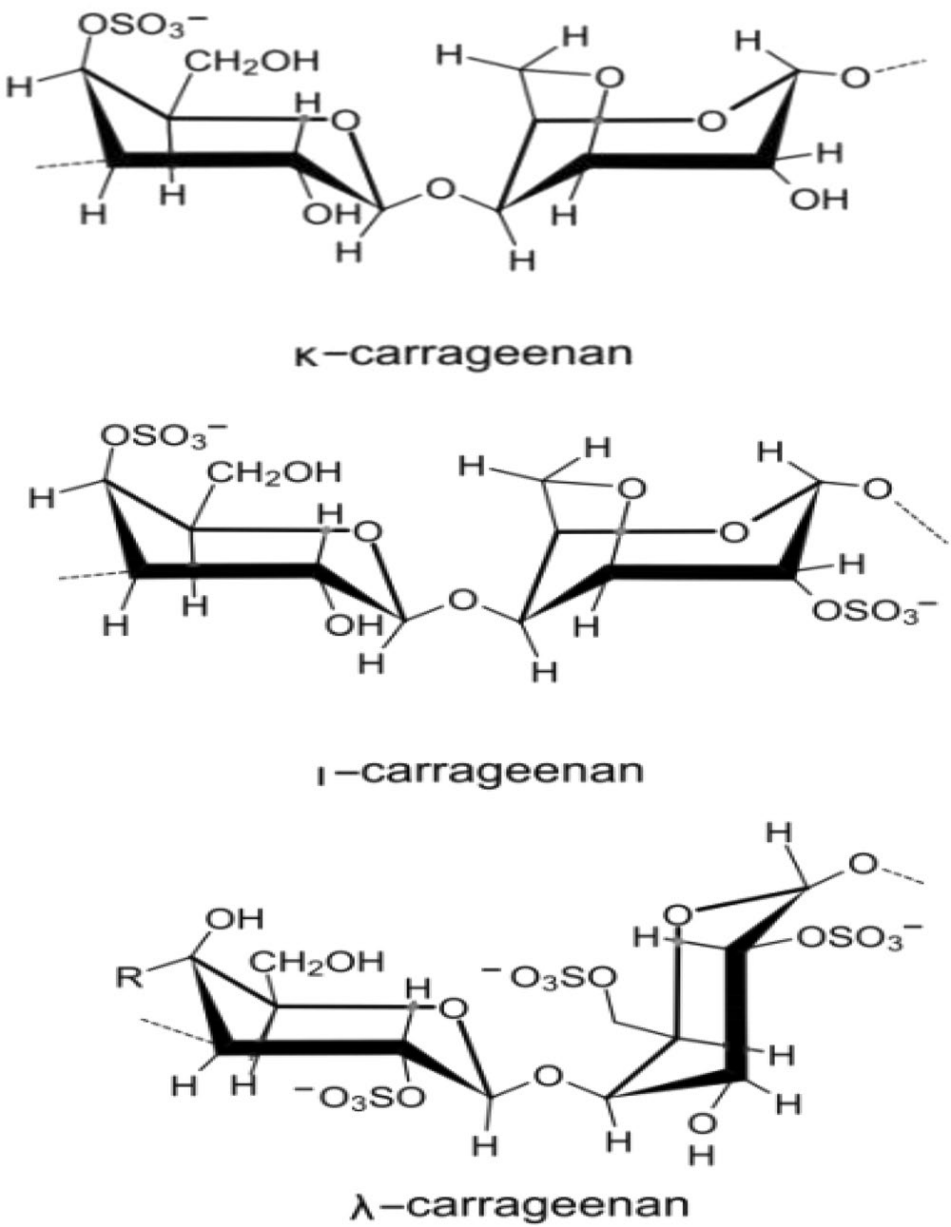
The chemical structures for three basic types of carrageenans.

Carrageenans are used in a variety of commercial applications because of their excellent physical properties, especially as thickening, stabilizing and gelling agents in dairy food products such as frozen desserts. Besides, carrageenan is also a very important component in pharmaceutical formulations and cosmetics^1–4^. Carrageenans are high molecular weight sulfated polysaccharides that composed of the repeating disaccharide units with alternating 3-linked β-D-galactopyranose and 4-linked α-galactopyranose or 3,6-anhydro-α-galactopyranose (AnGal)^5^. There are basically three types of industrially relevant carrageenans i.e. κ-(kappa), ι-(iota) and λ-(lambda) carrageenans (Fig. 1), which are classified according to their specific molecular structures, including their sulfation patterns and the presence or absence of 3,6-anhydro-α-galactopyranose on 4-linked α-galactopyranose^5,6^. κ-carrageenan’s molecular structure was reported as alternating 3-linked β-D-galactose 4-sulfate and 4-linked AnGal units. The ι-carrageenans have an additional sulfate group on C2(O) of the AnGal moiety, thus resulting in two sulfates per disaccharide repeating unit. The λ-carrageenans have three sulfate groups per disaccharide unit with the third sulfate group available at the C6 position of the 4-linked residue^5^.

The carrageenan content in food is usually around 0.01–1.00% of the total weight of food. In view of the complexities in chemical compositions of foods, they often require extensive sample preparation and purification procedures in order to eliminate interferences prior to analysis of carrageenan can be carried out^7–10^ using conventional methods such as chromatographic techniques^11–16^. Furthermore, methods such as precipitation followed by gravimetric sulphate quantification, necessitates a large amount of carrageenan samples in order to obtain sufficient quantity of sulphate for analysis^17^, and this makes rapid carrageenan analysis difficult.

Colorimetric techniques can also be applied for the determination of total carrageenans by using cationic dyes, which form complexes with carrageenan, and measured with an optical system e.g. spectrofluorometer or spectrophotometer. Colorimetric assay is sensitive towards the degree of sulphation, and it gives different responses towards different types of carrageenans^7^. Shirai and co-workers^18^ have revealed that metachromatic behaviour of methylene blue (MB) cationic dye, which induced by the polyanions was dependent on the type of interaction involved such as electrostatic interaction, hydrophobic interaction and interaction of π-electrons.

MB interacts electrostatically with carrageenan at low concentrations through cationic alkylamino functional group of MB and anionic ester sulphate group in the carrageenan molecule. This basic dye has been used since 1930 by Ewe^19^ for the identification and differentiation between carrageenan and other natural gums from plants. Graham^20^ used MB for quantitative assay of carrageenan. Soedjak^21^ reported a new strategy for colorimetric determination of carrageenans by using MB stain, and this method becomes the most useful one until the present time^1^. Apart from MB reagent, some other cationic dyes such as alcian blue^22^ and acridine orange^23^ have also been employed for the determination of carrageenan via colorimetric and spectrofluorometric methods, respectively. Electrochemical interrogation of carrageenan has been reported by using potentiometric titration technique. The electrochemical sensor was prepared by immobilizing dinonylnaphthalene sulfonate (DNNS) and tridodecylammonium chloride (TDMAC) ionophore in the polyurethane or poly(vinyl chloride) membrane containing *o*-nitrophenyloctylether (*o*-NPOE) plasticizer, and protamine or poly-L-arginine titrant was used as a titrant^24,25^. Recently, a new electrocatalytic analysis of carrageenans in seawater was reported based on the catalytic hydrogen evolution (CHE) reaction on the mercury electrode via adsorptive transfer constant current derivative chronopotentiometric stripping technique, whereby λ-, ι- and κ-carrageenans were distinguished based on the nature of their respective accelerated faradaic peaks at negative potential^26^.

**Figure 2:**
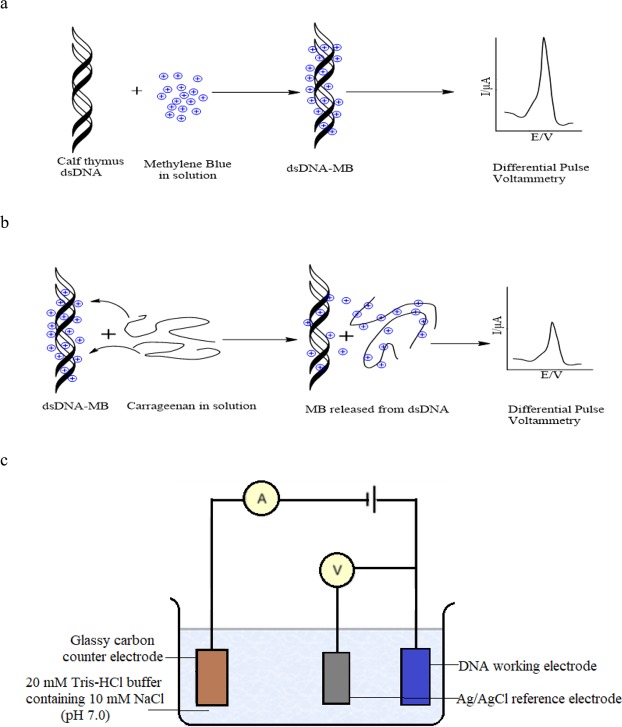
Schematic competitive electrostatic interaction of dsDNA and carrageenan towards binding with MB and electrical setup of the DNA device. (**a**) High DPV signal obtained after accumulation of MB in the dsDNA. (**b**) Lower DPV signal generated after immersion of the dsDNA electrode in the carrageenan solution. (**c**) The three-electrode system consists of a carbon SPE working electrode, Ag/AgCl reference electrode and a glassy carbon counter electrode.

The development of a variety of portable, rapid, and sensitive DNA biosensors have rapidly flourished for clinical, forensic, environmental and pharmaceutical applications due to their easy DNA testing procedures and providing immediate result on-the-spot^27–29^. In view of the innate net negative charge along the sugar-phosphate backbone of DNA molecule, and the well-established accumulation of MB with the double-stranded DNA (dsDNA), in this paper, we introduce a novel voltammetric DNA biosensor for carrageenan detection based on the competitive electrostatic interaction of dsDNA and carrageenan towards binding with MB. Figure 2 illustrates the schematic competitive binding of cationic MB to negatively charged dsDNA and carrageenan as well as the pictorial representation of the electrochemical voltammetric DNA device and the electrical setup involved.

The dsDNA of calf thymus was adsorbed on the screen-printed carbon paste electrode (SPE) at open-circuit potential, and intercalated with MB redox marker. Upon addition of carrageenan into the measurement cell, the cationic MB molecules released from the immobilized dsDNA due to the higher affinity of MB to form complex with carrageenan polyion. The decline in the reduction signal of MB with DNA biosensor was measured with differential pulse voltammetry (DPV). The proposed electrochemical DNA biosensor has potential for simple, sensitive and quick determination of carrageenan.

## Results and Discussion

### Investigation of Immobilized dsDNA Electrochemical Response

**Figure 3:**
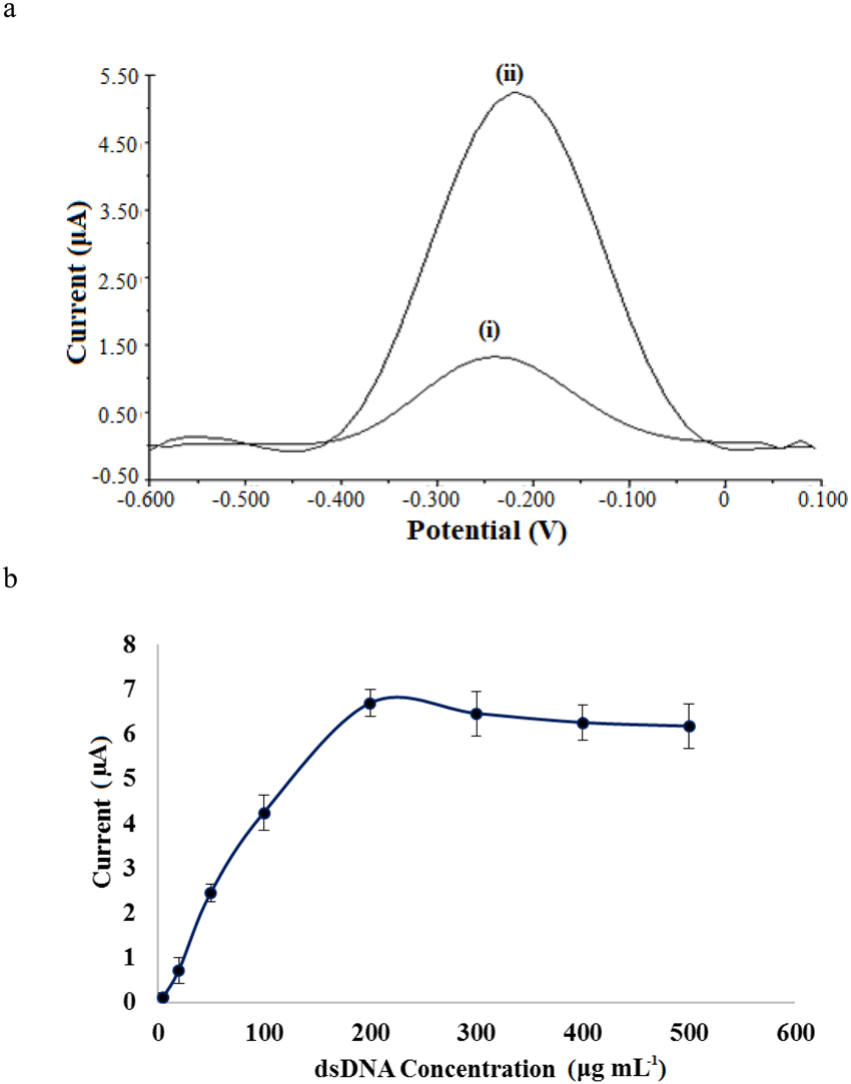
(**a**) Differential pulse voltammograms of (i) SPE and (ii) dsDNA-SPE in 20 mM Tris-HCl buffer containing 10 mM NaCl at pH 7.0 after accumulation with MB indicator for 300 s. (**b**) Effect of dsDNA loading on the MB-dsDNA-SPE response in a mixed 20 mM Tris-HCl/10 mM NaCl buffer at pH 7.0.

Both dsDNA-SPE and bare SPE current signals were measured with DPV after incubated with MB redox mediator, and a significant cathodic peak current (*i*_pc_) signal at −0.22 V was observed with dsDNA-SPE compared to the bare SPE (Fig. 3a) as the presence of immobilized dsDNAs possessed plenty of guanine bases for interaction with redox active MB^27^.

The DPV peak current increased sharply with a positive shifting of the DPV peak potential is typical for the intercalation of MB with dsDNA^30^. However, a small DPV peak current signal can also be perceived with the bare SPE, which could be due to the non-specific adsorption of MB redox indicator on the SPE surface. The increment in the DPV peak current signal of the dsDNA-SPE also indicates that the dsDNA has been successfully immobilized on the SPE surface.

Figure 3b shows the effect of dsDNA loading on the MB-dsDNA-SPE DPV response at −0.22 V. The biosensor response increased linearly from 5 µg mL^−1^ to 200 µg mL^−1^ dsDNA, and the MB *i*_pc_ signal reduced slightly thereafter until 500 µg mL^−1^ dsDNA loading. This was attributed to the over loading of dsDNA on the SPE, whereby the high degree of DNA hybridization rate has prevented the interaction between guanine bases and MB, thereby hindered the reduction of MB at the electrode surface.

### Accumulation of MB

**Figure 4:**
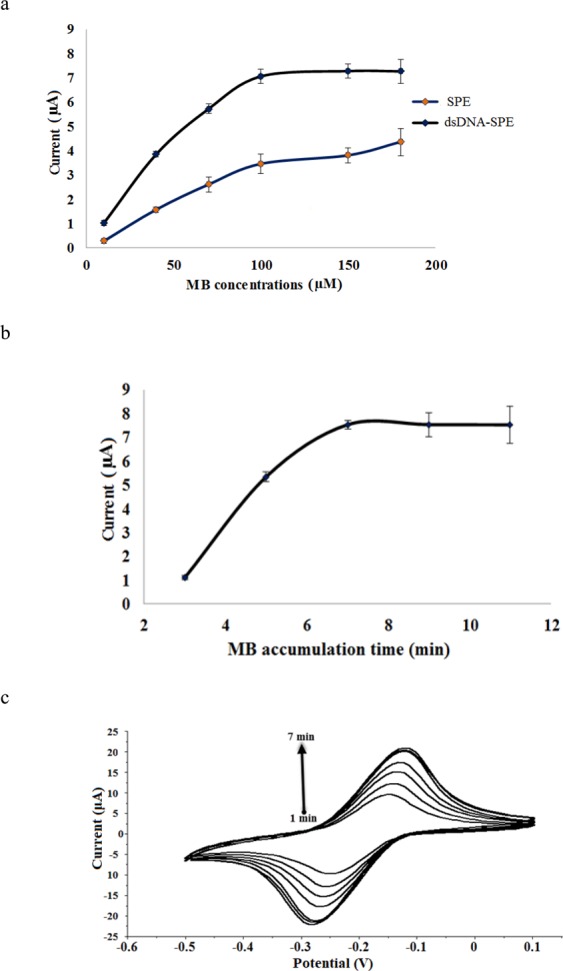
(**a**) Effect of MB concentration on the bare SPE and dsDNA-SPE response in 20 mM Tris-HCl buffer containing 10 mM NaCl (pH 7.0) with 7 min MB accumulation period. (**b**) DPV peak current signal of MB at dsDNS-SPE from 3-11 min MB accumulation periods. DPV response was measured in 20 mM Tris-HCl buffer in the presence of 10 mM NaCl at pH 7.0. (**c**) Cyclic voltammograms of dsDNA-SPE interacted with 100 µM MB at different time durations at a scan rate of 50 mV s^−1^.

The amount of MB used to intercalate with the immobilized dsDNA was found to influence the levels of DPV peak current signal. As Fig. 4a indicates, the MB *i*_pc_ signal increased with the increasing of the MB loading from 10–100 µM due to the increasing amount of guanine bases at the immobilized dsDNA were reacted with the increasing loading of MB.

Besides, the dsDNA-SPE was found to give one-fold greater response than the bare SPE with MB loading because of the available of guanine bases at the dsDNA-SPE for accumulation with the increasing loading of MB. Both electrodes showed saturated response with lower reproducibility performance especially for bare SPE when the MB was loaded above 100 µM. This might be due to the fact that the available guanine nucleotide binding sites at the immobilized dsDNA or the non-specific adsorption sites at the commercial carbon ink-based SPE have fully bound with the excess MB electroactive label.

In general, there are three different basic interaction mechanisms between MB and DNA, which have been identified to be (1) electrostatic interaction of cationic charge in MB with negatively charged phosphate backbone DNA, (2) MB intercalation within dsDNA double-helix (3) and MB binding with the exposed guanine bases on ssDNA^31^. Among the three ways of MB-DNA interactions, intercalation of MB into dsDNA gives the highest redox peak current signal^32^. The DPV peak current trending of the DNA-based biosensor following different MB accumulation durations is depicted in Fig. 4b.

The reduction signal of MB increased in the first 5 min as exemplified in the cyclic voltammograms of the dsDNA-SPE (Fig. 4c), which suggests an increasing guanine oxidation reaction rate at the immobilized dsDNA mediated by the MB redox intercalator with time. The intercalation reaction of MB with immobilized dsDNA was then reached to an equilibrium after 5 min of MB accumulation period^33,34^. Therefore, 5 min MB accumulation duration was optimum for a complete MB intercalation reaction with immobilized dsDNA.

### Effects of pH and Ionic Strength

**Figure 5:**
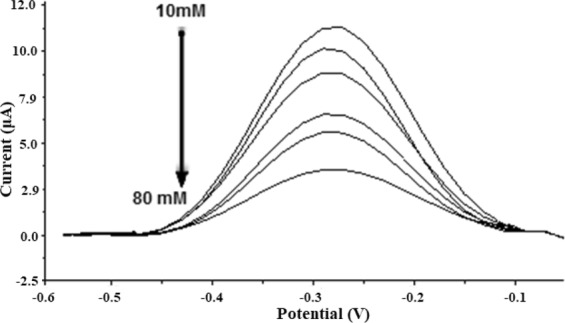
Effect of Na^+^ ion concentration on the MB *i*_*pc*_ response. DPV experiment was conducted on the MB-dsDNA-SPE in a mixed 20 mM Tris-HCl/10 mM NaCl electrolyte at pH 7.0.

It was found that the DPV peak current signal declined in both acidic and basic electrolyte solutions of Tris-HCl buffer. The maximum MB DPV *i*_*pc*_ signal was found with 20 mM Tris-HCl at pH 7.0. At higher or lower pH than pH 7.0, the hydrogen bonds between the nucleobases of DNA double-helix were broken up^35^, and that the MB intercalation reaction with the dsDNA cannot be occurred as a result of the DNA decomposition^36^. Therefore, 20 mM Tris-HCl at pH 7.0 was used as the electrolyte solution for all electrochemical measurements. Ionic strength of the electrolyte may affect the intercalation of DNA redox indicator with the dsDNA^37^. In the present study, low ionic strength electrolyte of 20 mM Tris-HCl buffer (pH 7.0) with 10 mM NaCl was noticed to render a more favourable intercalation of MB with the immobilized dsDNA (Fig. 5).

The addition of positively charged Na^+^ ion reduced the electrostatic repulsion between the negatively charged sugar-phosphate backbone of the dsDNA, thereby promoted the DNA hybridization reaction rate for subsequent intercalation with the MB redox label^34^. However, high ionic strength electrolyte solution at 20 mM NaCl and above introduced an ionic shielding to the negatively charged dsDNA sugar-phosphate backbone, which prevented the electrostatic interaction between the cationic MB and dsDNA. This has blocked the MB from reduction at the electrode surface, and that lower DPV peak current signal was obtained.

### DNA Biosensor Response towards Carrageenans and Polyanions

**Figure 6:**
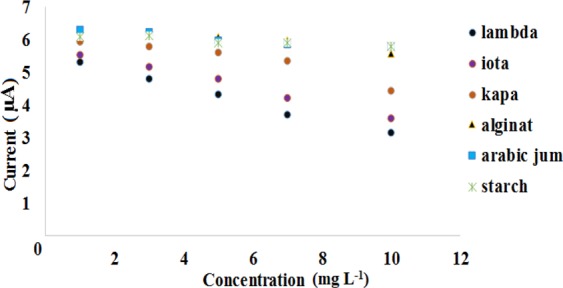
The DNA-based biosensor response towards various polyanions between concentration range of 1 mg L^−1^ and 10 mg L^−1^.

The optimized DNA-based biosensor has been tested with different types of carrageenans and polyanions in 20 mM Tris-HCl buffer containing 10 mM NaCl at pH 7.0. Based on the results shown in Fig. 6, the biosensor response declined proportionally with the increasing carrageenan concentration from 1–10 mg L^−1^.

This was attributed to the increasing released of the cationic MB redox indicator from the immobilized dsDNA for reaction with the respective carrageenans, and this has resulted lesser and lesser amount of MB being retained within the dsDNA-SPE, and that the MB reduction reaction rate at the electrode surface was getting reduced. It was noticed that the DNA-based biosensor demonstrated an overall lower DPV peak current response towards λ-carrageenan compared to ι- and κ-carrageenan due to the highest affinity of the λ-carrageenan for electrostatic binding with the MB based on 1:1 stoichiometry ratio between anionic sites of λ-carrageenan and cationic sites of MB dye molecule^21^. As the MB molecules released from the MB-dsDNA-SPE, it forms metachromatic complexes with the anionic λ-carrageenans. The dynamic linear concentration range of the DNA the biosensor between 1 mg L^−1^ and 10 mg L^−1^ carrageenan gave a regression coefficient (R^2^) at 0.98 with a limit of detection (LOD) of 0.08 mg L^−1^. The R-squared coefficient value was obtained from the linear line using format trend line without intercepting at zero coordinate.

κ-carrageenan has the lowest number of ester sulphate functional group, with only one sulphate group per disaccharide, thus the less anionic κ-carrageenan attracted less amounts of MB from the DNA electrode, and left behind a large amounts of immobilized MB within the dsDNA-SPE for mediation of guanine oxidation reaction on the SPE. Negligible response with the MB-dsDNA-SPE was acquired for other polyanions i.e. arabic gum, alginate and starch, whereby relatively constant MB *i*_*pc*_ signals were obtained in the biosensor working concentration range. This was because the MB molecules were remained intercalated in dsDNA on the electrode to mediate guanine oxidation response of the immobilized dsDNA as they do not selectively bind with those anionic polysaccharides i.e. starch, arabic gum and alginate. Therefore, it can be deduced that the interaction of the carrageenan with MB is governed by the sulphate content of the carrageenan^21,38,39^. Interaction between MB and polyanion is electrostatic, reversible, and stoichiometric. It is also interesting to note that the formation of metachromatic complex between carrageenan and MB was immediate without extended incubation duration was required compared to the other polyanions, and this simplified the analysis procedure for carrageenan determination^7,21^. Furthermore, the biosensor showed reproducible results with relative standard deviation (RSD) attained between 5.6% and 6.9% for three repetitive measurements of carrageenans.

The proposed competitive binding of MB with carrageenan by using the dsDNA electrode could sensitively detect the carrageenan at low levels compared to the previously reported electrochemical potentiometric titration techniques based on immobilized DNNS or TDMAC ionophore in the polymeric membranes with a dynamic linear range reported at 2–20 ppm and detection limit of 0.2 ppm^24,25^. The new adsorptive transfer constant current derivative chronopotentiometric stripping method for carrageenan detection demonstrated by Strmečki and Plavšić^26^ was only be able to selectively quantify the κ-carrageenan concentration in the mixture of three carrageenans, and may require the usage of separation technique prior to analyzing the mixture of different types of organic biomolecules. In addition, the detection limit of the chronopotentiometric stripping technique for carrageenan at >6 ppm cannot be applied for the low carrageenan concentration determination purposes.

### Determination of Carrageenan in Food Sample

Standard ι-carrageenan has been spiked into the pineapple jelly juice within the calibration concentration range of the biosensor for recovery of carrageenan in the jelly confectionery, and 20 mM Tris-HCl buffer (pH 7.0) was used to stabilize the pH level of the juice. From the results tabulated in Table 1, satisfactory recoveries between 106% and 110% imply that the voltammetric DNA-based biosensor based on competitive reaction of MB with dsDNA and carrageenan can be used for accurate and reliable carrageenan determination in the complex food samples especially in confectioneries.

**Table 1:** Recovery of ι-carrageenan in the edible pineapple jelly sample.

Carrageenan concentration spiked into the jelly sample (mg L^−1^)	Carrageenan concentration determined by DNA biosensor (mg L^−1^)	Recovery (%)
3.0	3.3	110
5.0	5.3	106
8.0	8.7	109

### Comparison of the DNA Biosensor Analytical Performance with Conventional Methods for Carrageenan Determination

Table 2 outlines the analytical performance of the developed electrochemical DNA biosensor and some other official methods commonly used for the assay of carrageenan in terms of detection limit, response time, simplicity and chemical consumption.

**Table 2:** Comparison of the DNA biosensor performance with chromatographic, spectrofluorimetric and colorimetric methods for evaluation of carrageenan.

Detection method	LOD	Response time	Simplicity	Chemical consumption	Reference
DNA biosensor	0.08 mg L^−1^	2 min	User friendly	Low	This work
Spectrofluorimetric	5.00 mg L^−1^	<1 h	Skillful operator	Low	Cundall *et al*.^23^
Gas chromatography	—	>1 h	Skillful operator	High	Lawrence & Iyengar^12^
High-performance anion-exchange chromatography	—	<1 h	Skillful operator	High	Jol *et al*.^15^
Colorimetric	0.05%	<1 h	Skillful operator	Low	Yabe *et al*.^22^

Gas chromatography and high-performance anion-exchange chromatography techniques which are sensitive for the determination of polysaccharides, however, they require elaborate hydrolysis step using acid e.g. trifluoroacetic acid in order to digest the polysaccharides into monosaccharides before determination of carrageenan can be done with the chromatographic methods. Furthermore, they involve long analysis time of close to an hour with high chemical consumption, and must be operated by trained personnel.

Evaluation of carrageenan with spectrofluorimetric and colorimetric methods, on the other hand, would require gravimetric precipitation process to be carried out followed by mixing with chemical absorption solvent such as monoethanolamine prior to optical measurement. This makes direct detection of carrageenan become impossible as aqueous amine solution is technically required following the precipitation step. As such, the proposed electrochemical DNA biosensor could provide promise to improve the analytical performance for quick, sensitive and convenient application with regard to direct determination of carrageenan without any pre-treatment of sample and derivatization.

## Methods

### Apparatus

DPV and cyclic voltammetry (CV) measurements were carried out by using AUTOLAB PGSTAT 12 potentiostat/galvanostat controlled by GPES 4.9 software (Eco Chemie, The Netherlands). The three-electrode system consists of a carbon SPE working electrode, which was purchased from Scrint Technology (M) Sdn Bhd, a Metrohm Ag/AgCl (3 M KCl) reference electrode and a glassy carbon counter electrode.

### Reagents and Materials

dsDNA stock solution was prepared by dissolving 5 mg of double-stranded calf thymus DNA powder (activated and lyophilized powder, catalog number 4522, Sigma) in TE buffer, which composed of 10 mM Tris-HCl (Acros Organics) and 1 mM EDTA (Duchefa Biochemie) at pH 8.0, and kept at 4 °C. Stock solution of methylene blue (MB, R & M Chemicals) at 1 mM was prepared in deionized water and stored at 4 °C in a dark environment. Analytical grade of ι-(iota) and λ-(lambda) carrageenans from Sigma, and κ-(kappa) carrageenan, calcium alginate, starch and gum arabic from Fluka were prepared in deionized water at 60 °C in a water bath followed by cooling to room temperature (25 °C), and diluted with deionized water to a final concentration of 100 µg mL^−1^. Dissolution of dsDNA, MB and carrageenan stock solutions were performed by using 20 mM Tris-HCl buffer and 10 mM NaCl (Sigma) at pH 7.0. Other chemicals used were of analytical reagent grade and all the solutions were prepared in Milli-Q deionized water (18 MOhm).

### Carrageenan Biosensor Fabrication

DNA immobilization was performed by depositing 3 μL of calf thymus dsDNA stock solution on the SPE and left to dry overnight at ambient conditions. The resulting dsDNA-modified SPE (dsDNA-SPE) was immersed in 20 mM Tris-HCl buffer for 5 min to remove the unbound dsDNA on the SPE electrode^40,41^. The dsDNA-SPE was then soaked in MB solution at open-circuit potential for 7 min, and rinsed again with copious amounts of 20 mM Tris-HCl buffer for another 10 s to remove those loosely bound MB from the DNA electrode. After that, the dsDNA-SPE modified with MB (MB-dsDNA-SPE) was immersed in the carrageenan solution for 2 min followed by rinsing with abundant amounts of 20 mM Tris-HCl buffer for 5 s before electrochemical scanning. Electrochemical measurement was carried out in 20 mM Tris-HCl buffer containing 10 mM NaCl at pH 7.0 between −0.6 and 0 V versus Ag/AgCl electrode with a scan rate at 0.005 V s-1 and step potential of 0.01 V.

### Optimization of Carrageenan DNA Biosensor

Several parameters were optimized before the DNA-based biosensor was used for carrageenan determination in edible jelly food sample. The amount of dsDNA loaded on the SPE was varied between 5 µg mL^−1^ and 200 µg mL^−1^ in order to obtain the highest DPV peak current response at −0.22 V in a mixed 20 mM Tris-HCl/10 mM NaCl buffer at pH 7.0. Optimization of MB loading on the dsDNA-SPE was carried out by varying the MB amount from 10–180 µM, whilst MB accumulation duration between 1 min and 9 min was investigated to achieve optimum biosensor performance using a constant dsDNA loading at 200 µg mL^−1^. The effect of electrolyte pH towards MB *i*_pc_ signal of the MB-dsDNA-SPE was examined by using a mixed 20 mM Tris-HCl/10 mM NaCl electrolyte solution between pH 2.0 and pH 11.0 with MB accumulation time was maintained at 5 min. Sodium ion (Na^+^) in the concentration range of 10–80 mM was used to adjust the ionic strength of 20 mM Tris-HCl electrolyte solution at pH 7.0. The DNA-based biosensor response against different types of carrageenans such as λ-, ι- and κ-carrageenans, and various polyanions including gum arabic, alginate and starch were studied by immersing the MB-dsDNA-SPE into the respective carrageenans or polyanions in 20 mM Tris-HCl buffer containing 10 mM NaCl at pH 7.0 for 2 min prior to DPV measurement at −0.22 V. Finally, recovery study was carried out by using pineapple jelly purchased from local hypermarket. The jelly was cut into pieces followed by blending 200 g of the jelly in 400 mL of deionized water. The pineapple pulp was then filtered away to obtain the clear juice and diluted with 20 mM Tris-HCl buffer (pH 7.0) to a final volume of 500 mL. Subsequently, about 250 mL of the juice was used to dissolve 0.3 mg of ι-carrageenan, and heated in a water bath at 60 °C for 15 min before left to cool to room temperature and stored in a refrigerator (4 °C).

## Conclusions

Biosensor determination of carrageenan by using DPV technique has been shown to be a simpler, faster and more sensitive method compared to the other previously reported chromatographic and spectrophotometric methods. The DNA-based biosensor is selective towards carrageenan detection via competitive electrostatic interaction of dsDNA and carrageenan towards MB redox label in the solution. DNA immobilization based on dry-adsorption method on the disposable SPE surface under open-circuit without any electrochemical pre-conditioning allows mass production of this electrochemical carrageenan biosensor in a short period of time at the lowest cost.

## Data Availability

All data are fully available without restriction.
